# Visual Speech Reduces Cognitive Effort as Measured by EEG Theta Power and Pupil Dilation

**DOI:** 10.1523/ENEURO.0288-25.2025

**Published:** 2025-11-06

**Authors:** Brian Kai Loong Man, Dorothea Wendt, Elaine Hoi Ning Ng, Kasper Eskelund, Tobias Andersen

**Affiliations:** ^1^Eriksholm Research Centre, Snekkersten 3070, Denmark; ^2^DTU Compute, Technical University of Denmark, Lygnby 2800, Denmark; ^3^Oticon A/S, Smørum 2765, Denmark; ^4^Department of Hearing Systems, Technical University of Denmark, Lygnby 2800, Denmark

**Keywords:** audiovisual speech, cognitive, EEG, listening effort, multisensory, pupillometry

## Abstract

Listening effort reflects the cognitive and motivational resources allocated to speech comprehension, particularly under challenging conditions. Visual cues are known to enhance speech perception, potentially by reducing the cognitive demands of the task. However, the neurophysiological mechanisms underlying this facilitation, especially in terms of effort-related changes, remain unclear. In this study, we combined pupillometry and electroencephalography (EEG) to investigate how visual speech cues modulate cognitive effort during speech recognition. Twenty-two participants (seven females) performed a speech-in-noise task under three modalities: (1) auditory-only, (2) audiovisual, and (3) visual-only. Task difficulty was manipulated via signal-to-noise ratio (SNR) in the first two modalities. Firstly, we found an inverted U-shape relationship between pupil dilation and frontal midline theta with SNR for audiovisual and auditory-only speech, consistent with prior models of effort allocation. Secondly, we observed the SNR at which the neurophysiological measures peaked was at a lower SNR for audiovisual speech. Surprisingly, we found pupil dilation to be larger overall in audiovisual speech, while frontal midline theta did not show differences in either modality. These findings highlight the complexity of interpreting physiological markers of effort and suggest that visual cues may alter the temporal dynamics or resource allocation strategies during speech processing. Our results support the extension of auditory-based models of listening effort to audiovisual contexts and underscore the value of integrating multimodal neurophysiological measures to better understand the cognitive and neural mechanisms of effortful listening.

## Significance Statement

Speech comprehension in noisy environments demands cognitive effort, which can be modulated by visual cues. Using pupillometry and EEG, we show that audiovisual speech shifts the effort-optimal signal-to-noise ratio and alters physiological markers of listening effort. These findings reveal that visual input reshapes resource allocation strategies during speech processing and support extending auditory-based models of effort to multisensory contexts. Our results underscore the value of multimodal neurophysiological approaches for understanding the neural mechanisms underlying effortful listening.

## Introduction

Many everyday conversations require the perception and integration of auditory and visual information. Cognitive resources are, however, limited (Kahneman and Tversky, 1973) and increasingly demanding conditions such as noise and hearing loss can induce higher cognitive demands and effort on speech perception (Peelle, 2017). Visual cues may alleviate such difficulties by providing additional articulatory information. Though intuitive, evidence for such an effect has been mixed (Mishra et al., 2013; Sommers and Phelps, 2016; Moradi et al., 2017; Brown and Strand, 2019; Gieshoff, 2021). Therefore, a direct comparison of listening effort under varying levels of task demands with auditory and audiovisual speech is needed to understand the effects of visual cues on listening effort.

The Framework of Effortful Listening (FUEL; Pichora-Fuller et al., 2016) is a theoretical framework that consists of two factors, Cognitive Demands and Motivation. The first factor refers to variables that influence the difficulty of a speech task. This may include extrinsic factors (external noise, reverberation, amplification, etc.) or intrinsic factors (hearing status, age, and working memory capacity; Rönnberg et al., 2013). The second factor comes from the Motivational Intensity Model (MIT; Richter et al., 2016). Effectively, a motivated individual would apply more effort as cognitive demands increased up to a certain level where maximal effort is exerted. When this level has been reached, the person will realize the increasingly poor trade-off between applying more effort and succeeding and becoming demotivated, leading to a reduction in observed effort. Indeed, such parabolic variation of effort has been observed repeatedly in experiments with different outcome measures reflecting cognitive effort such as reaction time and pupillometry (Sarampalis et al., 2009; Koelewijn et al., 2014; Ohlenforst et al., 2017, 2018; Wendt et al., 2017). Using an auditory-only speech paradigm, these studies observed the inverted U-shape, showing the effects of motivation and how it modulates whether effort increases or decreases with task demands.

Such a phenomenon, to our knowledge, has not been proven in the context of audiovisual speech. Therefore, the goal of this study is to take the findings stated earlier, where the focus was only on auditory-only speech, and investigate how effort changes for audiovisual speech across a wide range of task demands.

There are several physiological measures such as skin conductance and cortisol levels (McGarrigle et al., 2014) associated with effort allocation. Pupil dilation, specifically peak pupil dilation (PPD), is one such measure that we will use as our measure of effort. Pupillary responses are modulated by the locus ceruleus norepinephrine system (Gilzenrat et al., 2010) and an indicator of arousal during task performance. Its connection to processing load (Beatty and Wagoner, 1978) and thus effort has been supported by repeated observation of its sensitivity to changes in task difficulty such as lexical competition (Kuchinsky et al., 2013), noise (Koelewijin et al., 2012), and linguistic complexity (Piquado et al., 2010). Pupillometry has also been shown to be modulated by motivation in both visual (Wykowska et al., 2013) and auditory attention (Ohlenforst et al., 2018). Consequently, we expect PPD to follow an inverted U-shape as a function of signal-to-noise ratio (SNR) and for both auditory and audiovisual speech. Secondly, we also expect the inflection point (peak of inverted U-shape) to occur at a lower SNR for audiovisual speech due to the reduction in cognitive demands from visual cues (Peelle and Sommers, 2015).

In addition to PPD, we will also use EEG to measure effort allocation. Frontal midline theta has been shown to be related to high-level memory and cognitive process relating to top-down control (Cavangh and Frank, 2014). In the context of effort, two separate studies revealed frontal midline theta power to be correlated with subjective listening effort (Wisniewski et al., 2015) and that it decreases as the difficulty of the task becomes impossibly difficult (Wisniewski et al., 2017). Therefore, we expect similarities with PPD in terms of the morphology of the inverted U-shape, as well as shifting of the inflection point to a lower SNR for audiovisual speech due to lower cognitive demands (Peelle and Sommers, 2015).

Overall, this study investigates the effect of audiovisual speech on effort investment indicated by both pupil size as well as frontal midline theta. Firstly, we hypothesize that there will be an expected benefit of word recognition performance in AV compared with AO speech. Secondly, following the FUEL model (Pichora-Fuller et al., 2016), we predict both measures of cognitive effort (PPD and frontal midline theta power) to reveal the interaction between motivation and cognitive demands in the form of the inverted U-shape. Thirdly, we hypothesize, based on Peelle and Sommers (2015) predictions, that there would be a reduction in cognitive demands in AV speech. This will be revealed as a shift of the inverted U-shape toward a lower SNR for both pupil dilation and frontal midline theta.

## Materials and Methods

### Participants

Twenty-two adults (mean = 36.3 years, standard deviation = 11.6 years, 8 females) were recruited for the current study. All were native Danish speakers with no history of neural disorders or self-reported hearing problems. All were right-handed.

Each participant would take part in one experimental session that was divided into three parts. The study was approved by the ethics committee for the capital region of Denmark (journal number H-1-2011-033). The study was conducted according to the Declaration of Helsinki, and all the participants signed a written consent prior to the experiment and were financially compensated.

### Experimental environment

Participants were seated in a comfortable chair in a sound-attenuated booth. The chair was placed at a distance such that their heads were ∼70 cm away from the 27″ computer monitor (refresh rate 60 Hz). The experimental stimulation was controlled by a desktop computer running Psychtoolbox-3 in MATLAB and an external RME Fireface UCX sound card. Visual stimulation was controlled by the control computer outside the booth and presented in the monitor inside the booth. Auditory stimuli from a loudspeaker were positioned in front (0° azimuth) of the participant right above the monitor. Auditory noise stimuli were presented at a constant level of 70 dB SPL while speech levels varied between 58 and 70 dB SPL in steps of 4 dB. Luminosity inside the room was constant and set to be dim enough to allow headroom for pupil dilation while not being uncomfortably dark and monitored using a lux meter.

### Stimulus material

The Danish Sentence Test (DAST; Kressner et al., 2024) speech corpus was used as speech material. The DAST sentence lists contain both audio-only and audiovisual recordings for a broad range of sentences. The sentence material consists of short sentences between 4 and 5 s long and had varying syntactical structure that were also balanced across the lists. A female talker with minimal emotional facial expressions (known as talker F1 from DAST) was selected as speech material. Speech-shaped noise was created based on the speech material for the specific talker, where the phase was randomized across all sentences and averaged to create a stable white sounding noise. The auditory stimuli were sampled at 48 kHz while the visual stimulus was a 1,920 × 1,080 video recorded at 60 frames per second. It was also ensured that the luminosity of the visual speech stimulus would remain stable across all sentences and that the talker would remain still in the middle of the sentence.

### Experimental procedure

There were a total of nine conditions, spread between two modalities (auditory-only AO vs audiovisual AV) and four SNRs (−12, −8, −4, 0 dB), with an additional visual-only (VO) condition where no auditory speech is presented (2 modalities × 4 SNRs + VO = 9). SNRs have been chosen based on pilot testing indicating that this range corresponds to performances ranging between 0 and 100% across both modalities. Throughout all conditions, noise onset preceded the onset of acoustic speech by 2 s. The noise continued 2 s after speech offset.

**Figure 1.**
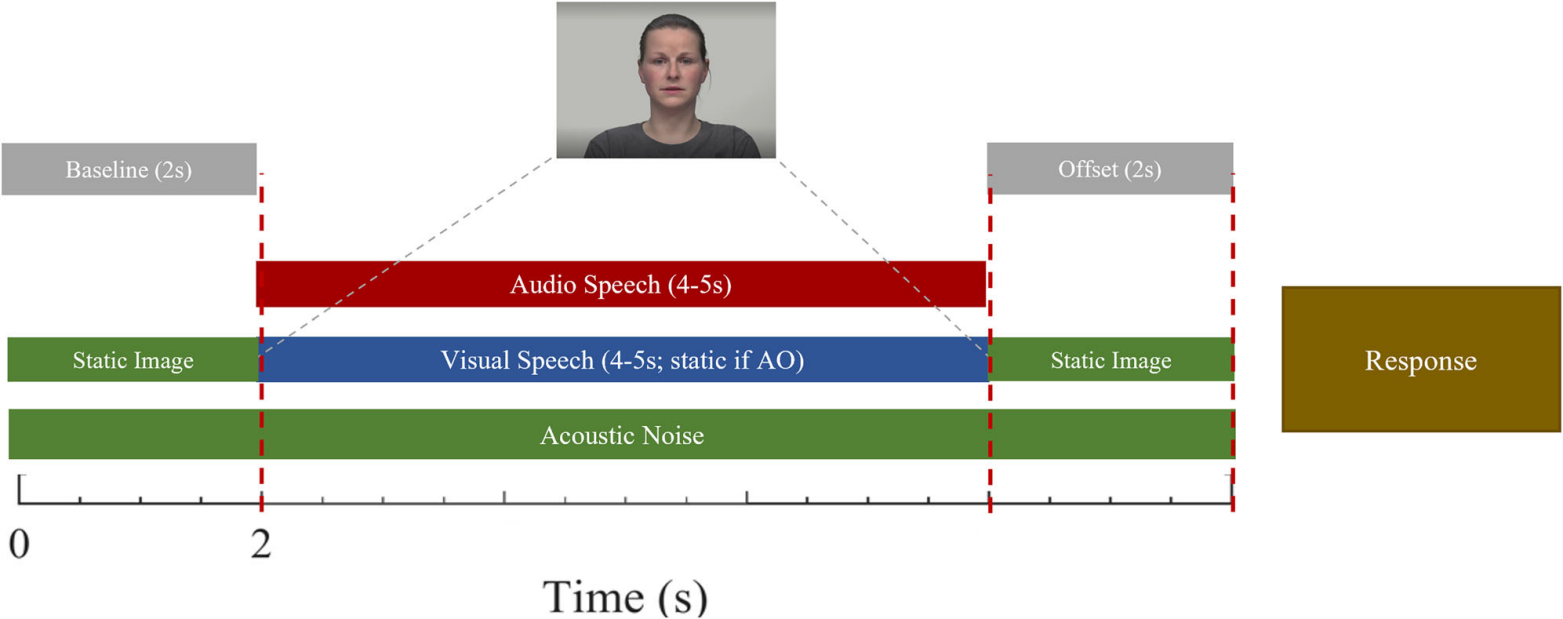
Experimental design.

For all modalities, a static image of the first video frame for the given sentence is presented for 2 s (see also Fig. 1) allowing adaptation to the image. This ensured similar sensory input across all conditions in all modalities. This would continue for 2 s and is done to ensure similar sensory input across all conditions in all modalities. The following presentation period would then differ based on the modality of the condition.

For the AV condition, the visual speech (video recording of the speaker) would start with sentence onset and is temporarily aligned with auditory speech (effectively the static image at the start would begin to move according to the visual speech). Once the speech finishes, the visual image will pause at the last frame of the speech video. For the AO condition, the visual image on the screen will remain static until the noise offset. Finally, for the VO condition, auditory speech instead is muted, leaving background noise and the video of the speech. After the speech presentation period, the visual speech would remain static again (last frame for AV and VO and same image for AO) while the acoustic noise continues for another 2 s before returning to a gray empty screen.

The experiment is divided into three blocks, of which each block refers to a given modality (AO, VO, or AV) with breaks in between each block to minimize fatigue. The order of the AO and AV blocks was randomized, and the VO condition was always the final block due to the possible influence of not knowing the talker’s voice. Participants were given 10 trials of practice for all three modalities before starting the actual experiment. Within the AO and AV blocks, the order of SNRs were randomized, such that participants would not know before a trial how difficult the trial would be.

The task across all three blocks for the participants was to pay attention to the speech signal and try to repeat to the best of their ability what they heard, in AO and AV conditions, or saw, in the VO condition. They were instructed to look at the talker’s face for all conditions. A fixation cross would appear at the noise offset to indicate that it is time to repeat what they heard. Scoring was based on repeating the three keywords from a sentence correctly. Therefore, a given trial would have a possibility of being scored as 0/3–3/3 for each given trial. The pacing of the experiment was determined by the test participant, as they could take as much time as they would like to repeat the sentence before pressing a button to proceed to the next trial. The total time taken for the experiment would last ∼2.5 h including breaks.

### Pupil data recording and preprocessing

Eye movements and pupil size of both eyes were continuously recorded using a Tobii Pro Spectrum Eye Tracker at a sampling rate of 1,200 Hz.

Pupillometry data were preprocessed and analyzed using MATLAB (MathWorks). To remove blinks, time points at which the size of the pupil were >3 standard deviations above or below the mean pupil size calculated over each trial were categorized as blinks and marked as invalid data in the time window 150 ms before and after the point of the identified blink. Subsequently, the missing data in the time series was linearly interpolated. Due to variable stimulus length (with constant baseline length), for some trials the pupil time series would contain portions after speech offset. Therefore, to not lose data from each trial, zero padding was used such that the whole pupil trace of each trial was embedded within each same-sized vector. Data was then low-pass filtered at 3 Hz (Butterworth, fifth-order filter). Trials were excluded if >40% of data points within a trial had to be interpolated. The entire dataset for a given participant was excluded from the analysis if >40% of trials had to be excluded (*N* = 1).

### Pupil data analysis

Baseline window was chosen to be the 1–2 s after noise onset where the static image was also presented. The first 0–1 s were omitted from the baseline window due to the startle effect caused by images and sounds appearing. The average dilation of this window was calculated and subtracted from the rest of the trial; for baseline correction, pupil size was averaged across trials separately for each condition and the maximum value for the average trace—the PPD was taken as the outcome measure.

### EEG data recording and preprocessing

EEG recordings were obtained using a 64-channel array (Biosemi) at a sampling rate of 1,024 Hz, referenced to both mastoid electrodes. EEG data were analyzed with EEGLAB (Delorme and Makeig, 2004), the FieldTrip toolbox (version 2022; Oostenveld et al., 2011), and custom scripts in MATLAB (MathWorks). Data were first rereferenced to the average across all electrodes, high-pass filtered at 0.5 Hz, and low-pass filtered at 60 Hz. Afterward, a 50 Hz band-stop filter was used to suppress main line noise. The traces were then downsampled to 256 Hz. Visual inspections of the raw recordings were performed and only one trial was removed from a single participant due to the absence of a trigger to signal the start of a trial. Independent components analysis (ICA) was calculated to remove independent components dominated by artifacts associated with blinks and electromyographic (EMG) activity. Components that could not be confidently explained were not removed. Therefore, an average of two components were removed for every given subject. Only one channel (AF3) was removed and interpolated from neighboring channels for a specific participant due to systematically large voltage values that could not be attributed to EEG activity. All artifacts were identified through visual inspection. The recordings were then epoched and divided into trials that start from the noise onset at 0 s to 7 s after. This would mean that each trial would be 7 s in length, where the first 2 s are the baseline period while the remaining 4 s were active speech listening. The data was trimmed this way due to varying trial lengths where the shortest speech sequence was 3.87 s (up to 5.4 s).

### Time–frequency analysis of EEG

Oscillatory activity for theta power (4–7 Hz) were obtained by convolving single-trial EEG signals with Morlet wavelets with varying cycle widths (baseline 3 cycles) in the time domain, with frequencies between 3 and 13 Hz linearly spaced 0.5 Hz apart, from 0 to 6 s across all electrodes and participants. The full-width at half-maximum (FWHM) ranged from 109 to 172 ms with increasing wavelet peak frequency. After obtaining power values for each trace, baseline correction was performed by taking the average power of 0–2 s and dividing each sample from 2 to 6 s with the baseline power. The power values were subsequently log transformed to obtain decibel (dB) values and averaged across the time window to obtain the mean log power over the active listening period. Since, on the scalp level, we were interested in frontal midline theta power, channel-level power would consist of channels AF3, AF4, and AFz.

### Statistical analysis

No data from the behavioral results were discarded. Therefore, leading to a total of 22 participants, each with a single observation of percentage keywords correct for one of the eight given conditions (8 × 22 = 176 observations). The visual-only condition was not included as a factor in the statistical models. Pupil data selection and cleaning were applied to the data from 21 participants after one participant was excluded due to having too many invalid trials from equipment failure. No subjects were excluded for EEG data, resulting in 22 observational units. Linear mixed models (LMMs) were applied to analyze the data. One observation of PPD was recorded for a given SNR and modality for each participant, resulting in a total of 8 × 21 = 168 observations. Similarly, due to no subject exclusion, 8 × 22 = 176 observations were obtained for EEG data. EEG data consisted of theta power values at frontal channels. Pupil dilation and EEG frontal midline theta power were both independently averaged to provide a single value per condition for each participant. This averaging is commonly done in pupillometry and EEG literature (Zekveld et al., 2011; Koelewijn et al., 2014; Wendt et al., 2017; Ohlenforst et al., 2018; Seifi ala et al., 2020; Kraus et al., 2023).

Statistical analysis was conducted using R-studio with the lme4 library (Bates et al., 2015) and statistical tests were performed using lmerTest (Kuznetsova et al., 2017). LMMs were constructed for each outcome measure. The first was the from the word recognition results; the model would thus consist of the factors for Modality (AO, AV) and SNR (−12, −8, −4, 0 dB SNR), including the interaction between the two factors (Modality:SNR). This was followed by two more models for PPD and frontal midline theta. Since both are measures of listening effort, we hypothesized they would follow a quadratic relationship with task demands (SNR). Thus, an additional quadratic term for SNR $\left(\mathrm{SN}R^{2}\right)$ was introduced in the model for these two models as well as the interaction between the fixed effects. Note that the VO condition is not included in the statistical models. Across all models mentioned above, subjects’ identity was treated as a random effect, meaning that all the models were linear mixed effect models (LMM). Model diagnostics were performed on all four models. To investigate and quantify the degree of the shift in the peak where maximal PPD and frontal midline theta power is observed, we also implement a posterior simulation of the model coefficients to estimate the peak for each given modality. This was performed by the sim() function from the “arm” package (Gelman and Su, 2024). This function generates samples from the approximated posterior distribution of the model coefficients estimated from the LMMs, with covariance derived from the covariance matrix. We took 1,000 total draws from each model coefficient ( SNR, $\mathrm{SN}R^{2}$, Modality, Modality:SNR; $\mathrm{Modality}:\mathrm{SN}R^{2}$). Based on the assumption that a quadratic function is fitted, we used the extracted coefficients to compute the vertex (peak SNR) at which PPD and frontal midline theta power:

$$\mathrm{Pea}k_{\mathrm{AO}}=-\frac{\beta_{\mathrm{SNR}}}{2\beta_{\mathrm{SN}R^{2}}},$$

$$\mathrm{Pea}k_{\mathrm{AV}}=-\frac{\beta_{\mathrm{SNR}}+\beta_{\mathrm{Modality}:\mathrm{SNR}}}{2(\beta_{\mathrm{SN}R^{2}}+\beta_{\mathrm{Modality}:\mathrm{SN}R^{2}})}.$$

This results in 1,000 samples for each of the simulated peaks from where we can compare the peaks between the two modalities. Consequently, we carried out a one-sided paired *t* test to evaluate whether the peak SNR in the auditory condition was greater than the audiovisual condition.

Finally, models with quadratic terms initially demonstrated high collinearity between the linear SNR and quadratic SNR terms, potentially leading to convergence and stability issues. Therefore, scaling was performed such that the mean SNR (−6 dB) was deducted from all levels of SNR to eliminate collinearity while preserving the same amount of variance. As a result, the two terms were not correlated in the final models.

### Data and code availability

All code/software and data described in the paper used to generate this manuscript and figures are available at https://github.com/brianman515/PhD-Study1.

10.1523/ENEURO.0288-25.2025.d1 Data 1 GitHub link (https://github.com/brianman515/PhD-Study1) contains all files used for the preprocessing of behavioral, EEG, and pupillometry recordings. All analysis scripts are contained within tools/, which are further divided into the behave/, eeg/, and pupil/ subfolders respectively. cofig/ contains the necessary configuration files that support the analysis. tools/stats.Rmd is the statistical analysis script that produced and evaluated all statistical models mentioned in the manuscript. Download Data 1, ZIP file.

## Results

### Better behavioral performance for audiovisual speech compared with audio-only speech

The behavior performance is displayed in Figure 3. The sentence recognition scores across SNRs are shown by the dotted lines, where the color of the lines represents the modality of the condition.

The LMM revealed significant main effects of SNR (*p* < 0.0001, *F*_(1,151)_ = 2,805, *β* = 0.078, SE = 0.002), Modality (*p* < 0.0001, *F*_(1,151)_ = 121, *β* = 0.136, SE = 0.012), and interaction between the two factors (*p* < 0.01, *F*_(1,151)_ = 10.3, *β* = −0.01, SE = 0.003), indicating that sentence recognition is affected by differences in the listening conditions (modality and SNR).

### Pupil dilation reflects cognitive effort for both modalities and lower effort for audiovisual speech

**Figure 2.**
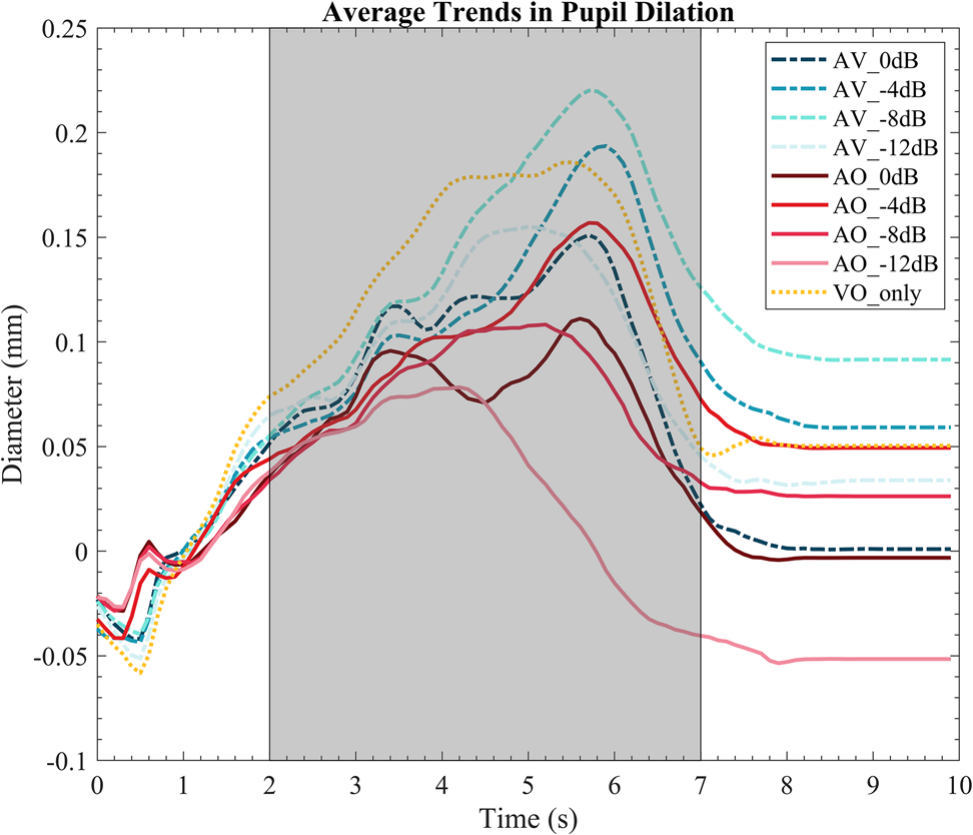
Time course of pupil dilation. The gray area reflects the time windows of interest for statistical analysis (2–6 s). The speech starts at *t* = 2 s.

Figure 2 plots the time course of pupil dilation for all conditions. The gray area marks the time window of interest, where PPD was obtained between the 2 and 7 s time window. Qualitatively, it can be observed that pupil size increased from baseline period (0–2 s time window) for most conditions, reaching their PPD at the later part of the stimulus (indicated by gray area, 2–7 s time window).

The LMM revealed significant effects for the quadratic term SNR (*p*_snr^2_ < 0.001, *F*_(1,142)_ = 11.4, *β* = −0.001, SE = 0.0005), indicating parabolic relationship between SNR and PPD. The effect of modality was significant (*p*_modality_ = 0.00574, *F*_(1,142)_ = 7.87, *β* = 0.05, SE = 0.02). The linear fixed effect of SNR was not significant (*p*_snr_ = 0.388, *F*_(1,142)_ = 0.750, *β* = 0.003, SE = 0.002). Importantly, there was a significant interaction between the linear SNR term and modality (*p*_modality:snr_ = 0.0403, *F*_(1,142)_ = 4.28, *β* = −0.005, SE = 0.002), indicating that the PPD across SNRs varied between the modalities. Finally, there was no significant interaction between the quadratic SNR term and modality (*p*_modalty:snr^2_ = 0.786, *F*_(1,142)_ = 0.737, *β* = 2 × 10^−4^, SE = 1 × 10^−4^).

**Figure 3.**
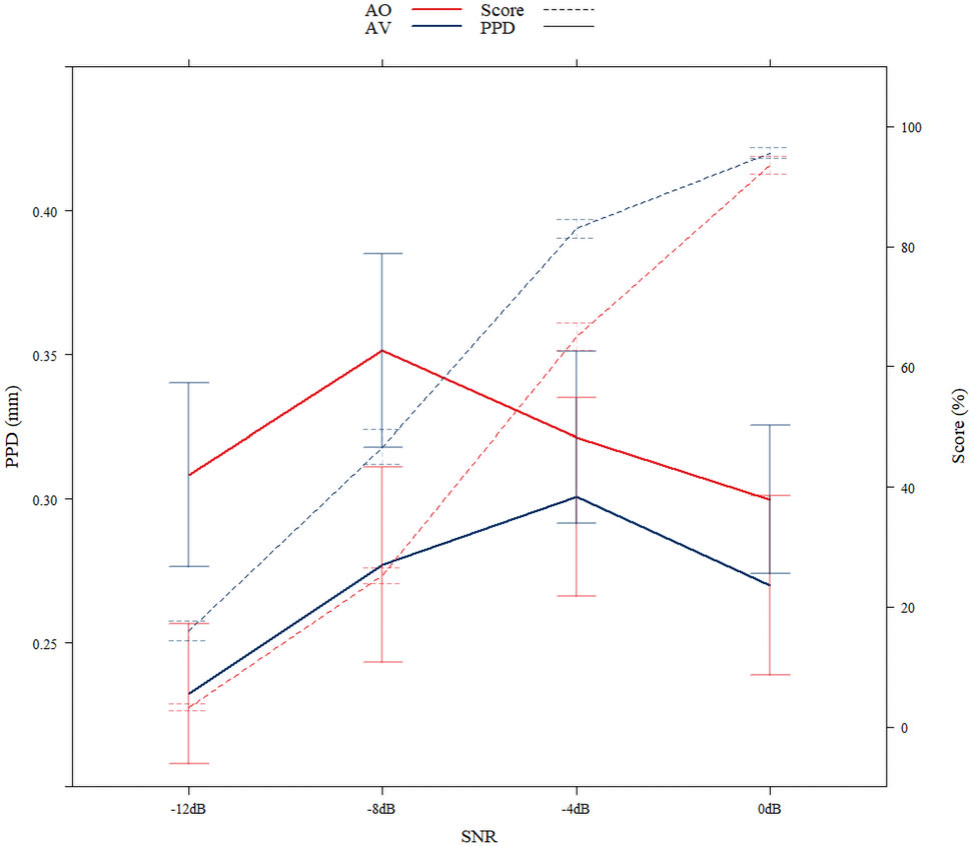
Behavioral performance and peak pupil dilation. Results from behavioral performance (dotted lines, percentage keywords correct) and pupillometry (solid lines, peak pupil dilation). Auditory-only (AO) and audiovisual (AV) modalities are reflected by red and blue, respectively. Error bars represent the standard error of the mean.

Figure 3 shows the PPD data averaged across participants for the two modalities (AO, red; AV, blue) and the four SNR levels. At least three observations can be made here: firstly, PPD is significantly higher for AV speech compared with AO speech. Secondly, both modalities demonstrate the statistically significant quadratic relationship with SNR, where the PPD would increase initially as SNR decreased before reaching a peak and decreasing again. For the AO condition, the participants had a peak PPD at −4 dB SNR, where they achieved ∼50% correct recognition. In contrast, the AV condition instead showed peak PPD at −8 dB SNR, where they achieved a similar correct recognition at ∼50%. Such a shift in the peak reflects the significant interaction between the SNR and modality. The peaks also seem to lie close to the 50% correct point of sentence recognition scores.

**Figure 4.**
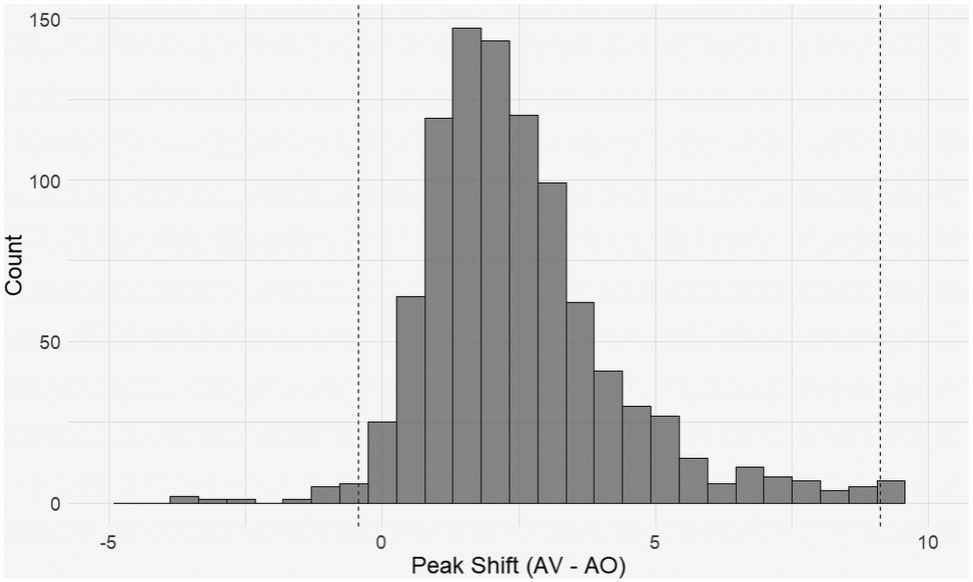
Distribution of the peak shifts (AV-AO). Dashed lines indicate 95% confidence intervals.

To further support our hypothesis that the peak SNR would be observed at a lower SNR for the AV modality, our posterior simulation method yielded the following distributions for the estimated SNRs at which PPD was observed for either modality (Fig. 4).

There is a clear difference in the obtained vertices for either modality with a mean shift of 2.6 dB. One-sided *t* tests with the alternative hypothesis that the peak SNR for the AO condition is greater than AV was statistically significant (*t* = 32.0, *p* < 0.01, 95% CI = [−0.46, 8.74]).

### Frontal midline theta power reflects changes in cognitive effort

**Figure 5.**
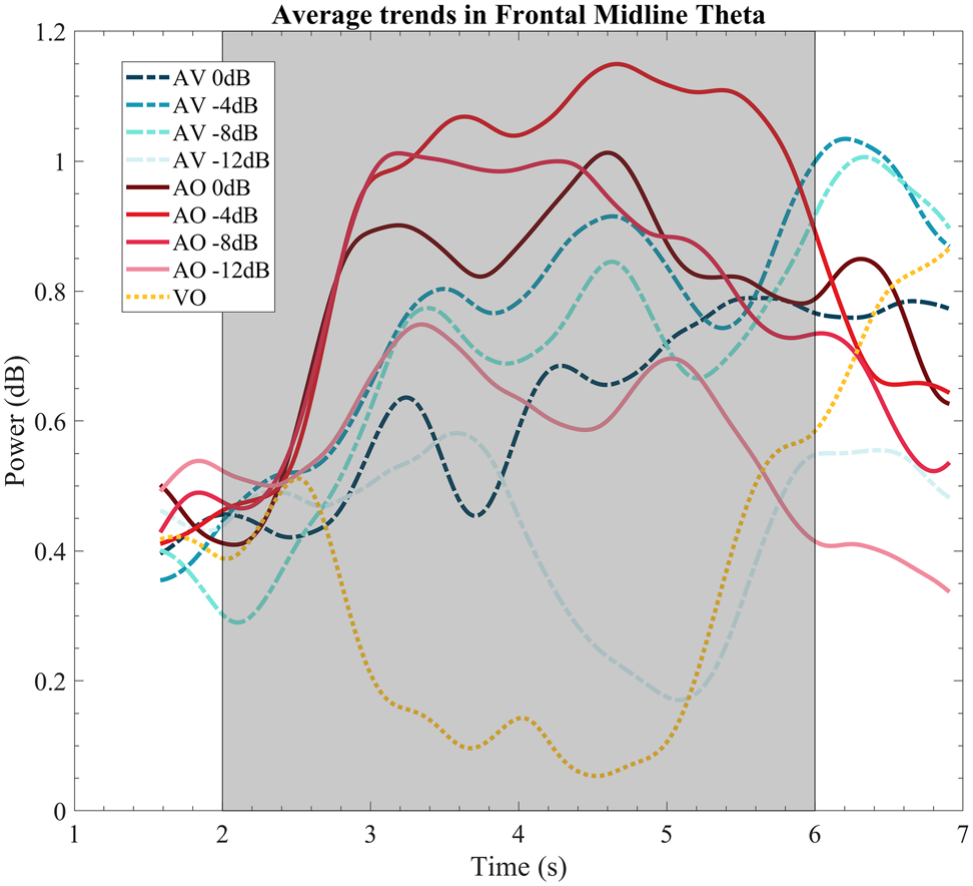
EEG band power time courses for frontal midline theta. Gray areas reflect the time windows of interest for statistical analysis (2–6 s). The speech starts at *t* = 2 s.

Figure 5 shows the time course for frontal midline theta power in the respective frontal channels.

**Figure 6.**
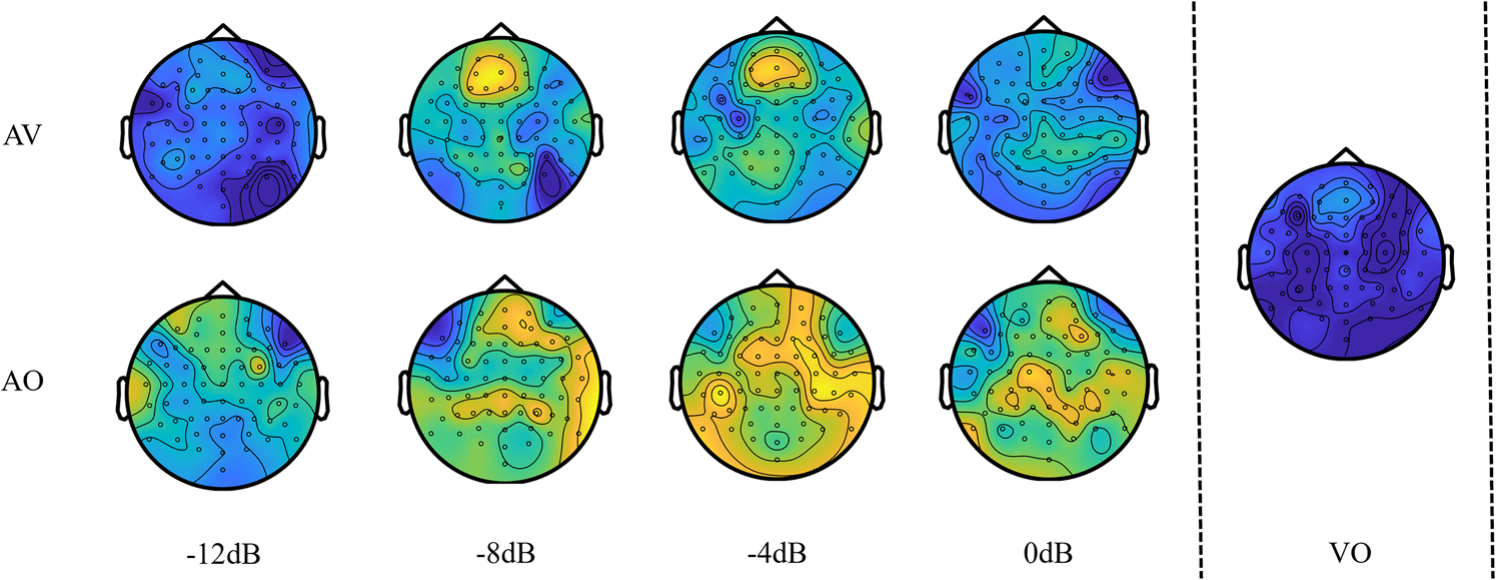
Topographical maps obtained after time-averaging the band power for theta. Strong midline-frontal theta power can be observed for AV speech at −4 dB, −8 dB and to a degree for −12 dB and VO speech. It is less prominent but still observable in AO speech. For AO speech, the theta power seems to be strongest at −4 dB and weakest at −12 dB.

According to the figure, it can be observed that the onset of the stimulus caused a short spike in activity across all conditions for the first second of onset (*t* = 2–3 s). They would then reach some level of stability for the rest of the speech stimulus before decreasing again after stimulus offset (*t* = 6 s). The scalp map (Fig. 6) shows a clear and distinct frontal midline theta, especially at −4 and −8 dB SNR for the AV condition, while the AO condition showed a more diffused activation across the scalp with less specificity at the frontal midline region.

**Figure 7.**
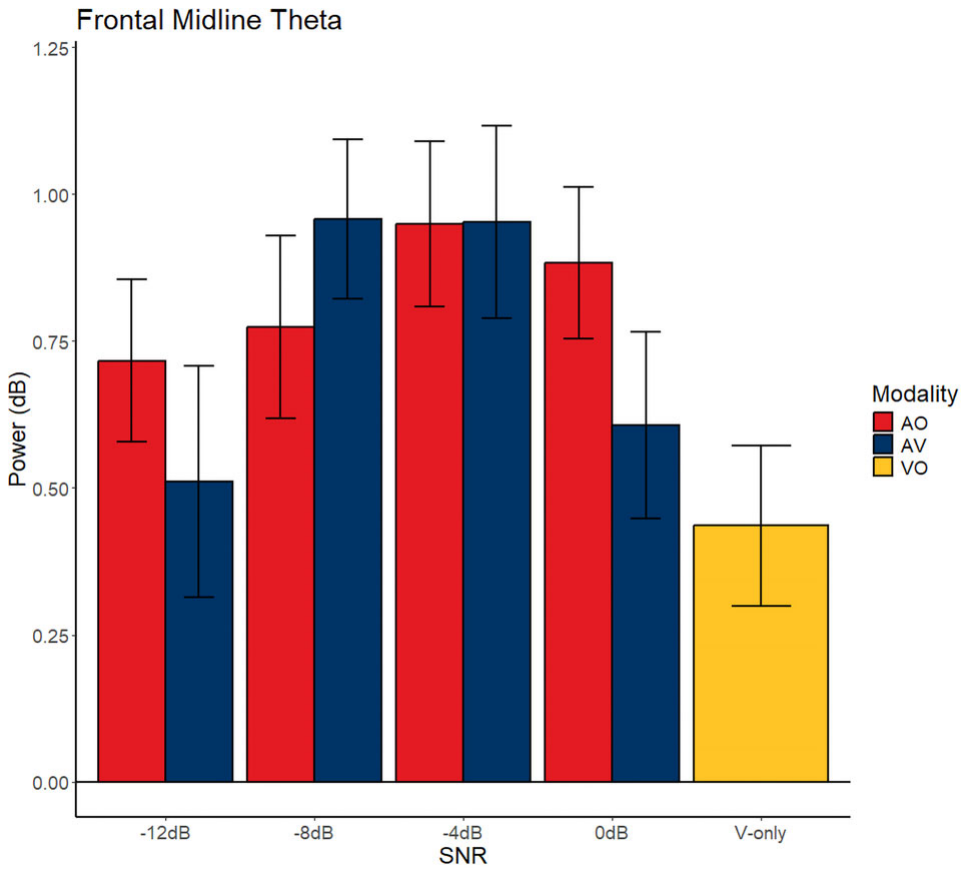
Band-averaged power frontal midline theta power. Red, blue, and yellow colors indicate the conditions AO, AV, and VO, respectively. Gray dots on the scalp maps indicate the electrode array of interest from where power was extracted. The error bars indicate the standard error of the mean (SEM).

Figure 7 shows the band-averaged frontal midline theta power for all conditions: the band power was not statistically significant between the modalities as shown by a nonsignificant fixed effect of modality (*p*_modality_ = 0.284, *F*_(1,149)_ = 1.16, *β*= 0.135, SE = 0.125). Another more observable effect shown by the figure is that frontal midline theta power seems to vary with SNR levels which is supported by a significant effect of the quadratic term SNR (*p*_snr^2_ = 0.004, *F*_(1,149)_ = 8.53, *β* = −0.002, SE = 0.003). There was no significant effect of the linear SNR term (*p*_snr_ = 0.174, *F*_(1,149)_ = 1.86, *β* = 0.02, SE = 0.01) while there was a significant interaction of the quadratic SNR term and modality (*p*_modality:snr^2_ = 0.0347, *F*_(1,149)_ = 4.54, *β* = −0.01, SE = 0.005). The interaction of SNR and modality was not significant (*p*_modality:snr_ = 0.320, *F*_(1,149)_ = 0.573, *β* = −0.001, SE = 0.02).

Finally, the shape of the AV and AO curves for frontal midline theta resembles that of PPD (Fig. 3), especially at the SNR where the maximum power was observed. The significant interaction between the modality and SNR, much like pupil dilation, also suggests a shift in the peak of the AV curve toward the left.

**Figure 8.**
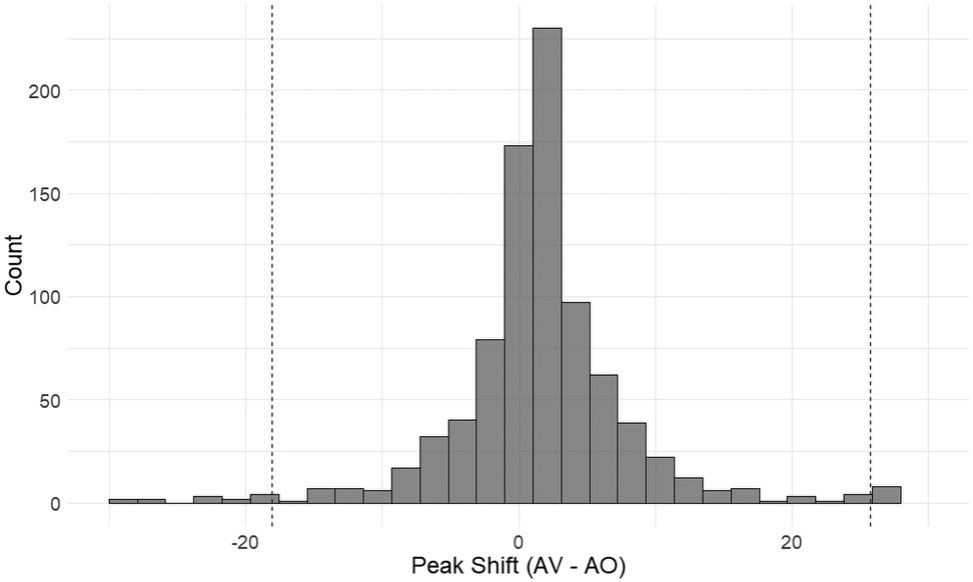
Distribution of the peak shifts (AV-AO). Dashed lines indicate 95% confidence intervals. Dashed lines indicate the 95% confidence intervals.

Again, we carry out the same posterior simulation method to quantify the degree of peak shift between the modalities:

Figure 8 shows peak shifts with more bias toward a positive shift. Again, one-sided *t* tests with the alternative hypothesis that the peak SNR for the AO condition is greater than AV was statistically significant (*t* = 1.65, *p* < 0.05, CI = [−25.2, 24.6]) with an average shift of 0.86 dB.

## Discussion

In the current study we investigated how two commonly used neurophysiological measures of cognitive effort change with task demands and stimulus modalities. Behavioral measures showed an expected benefit of visual cues as revealed by higher performance levels for AV speech compared with AO speech. For pupil dilation, we also had three main observations. First, we observed an inverted U-shape for pupil dilation across SNRs for both modalities indicating changes in cognitive effort. More specifically, a significant quadratic term for SNR was observed, indicating a parabolic relationship and therefore an inflection point of peak PPD was observed for both modalities. This finding is aligned with previous work on listening effort for audio-only stimuli (Wendt et al., 2017; Ohlenforst et al., 2018). Secondly, there is evidence for a reduction in cognitive demands for AV speech as the inflection point was observed at a lower SNR for AV compared with AO speech. This was supported by the significant interaction term between Modality and SNR for both neurophysiological measures. We further support this by carrying out a posterior simulation procedure and quantified the peak shift to be around the mean of 2.6 and 0.86 dB for pupil dilation and frontal midline theta respectively. Finally, pupil dilation was significantly higher in AV speech compared with AO speech, and the same was not observed in frontal midline theta.

In the following, it will be discussed how the improved AV performance in combination with the inverted U-shape observations for pupil dilation and frontal midline theta can be interpreted in the context of effort allocation in audiovisual speech processing based on existing theories of cognitive effort during speech perception including the FUEL model. We will also present evidence that supports the argument that visual cues indeed reduce cognitive demands during speech perception (Peelle and Sommers, 2015). Finally, we discuss the surprising misalignment of modality effects between the two measures of cognitive effort and how the measures may be modulated by different factors beyond effort.

### Pupil size and frontal midline theta both reflect deliberate effort allocation during speech perception

Effortful listening is defined as the deliberate allocation of mental resources to overcome obstacles in goal pursuit when carrying out a task (Pichora-Fuller et al., 2016). Pupil dilation is one such measure, due to a strong body of evidence where an increase in pupil size would be observed with higher cognitive demand regardless of whether a given task was auditory or visual (Zekveld et al., 2010; Koelewijn et al., 2012; Zekveld and Kramer, 2014; Winn et al., 2018; Kadem et al., 2020; Martin et al., 2020; Stolte et al., 2020; Kraus et al., 2023). Additionally, pupil dilation captures the interaction between cognitive demands and motivation in the form of the inverted U-shape (Ohlenforst et al., 2017, 2018; Wendt et al., 2017), thus indicating the deliberate aspect of effortful listening.

On the other hand, frontal midline theta has also been linked to cognitive effort and control (Cavanagh and Frank, 2014). Such modulations in power due to effort has been generalized to nonauditory tasks (Gevins et al., 1997; Jensen and Tesche, 2002; Onton et al., 2005). Frontal midline theta power has also been shown to be active during high-level cognitive memory processes including memory encoding and retrieval, retention, and realizing for top-down modulation (Jacobs et al., 2006; Raghavachari et al., 2006). In their study, Wisniewski et al. (2015) showed increasing frontal midline theta power (Fz) with SNR during the speech presentation interval. Despite a wide range of SNRs (−12 to +12 dB) this effect was linear. This however could be explained by the narrow range of performance levels (0.49–0.99 correct word recognition). Instead, they found subjective effort to increase with lower SNRs, and it to be positively correlated with frontal midline theta power, hence arriving to its association with listening effort. A later study by Wisniewski et al. (2017) showed frontal midline theta power to increase with difficulty initially in an auditory task; the power would then decrease when the task became impossibly difficult. This observation could indicate listeners giving up on the task, thus indicative of an inverted U-shape. However there were only three difficulty levels (easy, difficult, impossible) and the quadratic relationship was not statistically verified.

Given that both measures (pupil dilation and frontal midline theta power) have been shown to change with cognitive demands and have been linked to memory processes, it suggests that both can potentially be used as an indicator of effort allocation. Importantly, both measures have demonstrated a reduction when a task becomes too difficult and the participant disengages with the task. This inverted U-shape is a generally accepted indicator of cognitive effort for results that include a wide range of performance levels (Zekveld and Kramer, 2014; Pichora-Fuller et al., 2016; Ohlenforst et al., 2017, 2018; Wendt et al., 2017; Wisniewski et al., 2017; Seifi Ala et al., 2020) and is evidence of the interaction between cognitive demands and motivation. However, it was not known whether such a feature would extend to multisensory speech. Here, we report its presence for both pupil dilation and frontal midline theta. This indicates that both measures captured the parabolic relationship between cognitive effort and task demands, extending the FUEL to multisensory speech. The findings thus show the dynamic utilization of working memory resources during listening (Rönnberg et al., 2013).

### Congruent visual cues reduce cognitive demands during speech perception

The FUEL model states that variations in cognitive effort is attributable to two factors—cognitive demands and motivation (Pichora-Fuller et al., 2016). According to Peelle and Sommers (2015), the addition of visual cues reduces the cognitive demands of a speech perception task via two mechanisms, namely, spatiotemporal correlations and constraining of lexical neighbors. Visual speech is shown to be correlated with auditory speech (Chandrasekaran et al., 2009), allowing temporal prediction of phonemes. Indeed, participants were more able to detect tones presented in both regular and irregular rhythmic intervals during audiovisual presentation instead of auditory-only presentation (ten Oever et al., 2014), showing the benefit of spatiotemporal correlations. On the other hand, visual speech is also influenced by the neighborhood framework in which viseme functions as the basic perceptual unit. During speech perception, units of phonemes and visemes are continuously partitioned as perceptual units defined by their respective features. Target words that are close to many perceptually close neighbors will be harder to detect as their features overlap with the competition. Tye-Murray et al. (2007) presented the benefit of visual cues in a word identification task, showing the benefit to be related to visual cues constraining the lexical competition when compared with unimodal speech. In our results, not only did we see a shift in the peak of the inverted U-shape curves for both pupil dilation and frontal midline theta, the shifting of the curves also coincided with the general improvements across all SNR levels with maximal effort exerted around the 50% performance level. Additionally, we support this argument with a separate posterior simulation process. Using this method, we show that pupil dilation and frontal midline theta power to peak at a lower SNR for audiovisual speech. The models quantify the shifting effect to be 2.6 and 0.86 dB, respectively. This indicates that, due to the mechanisms mentioned above, less cognitive effort is needed to resolve the task at a given SNR when there are visual cues. Combined, both PPD and frontal midline theta lend evidence to the prediction of reduced cognitive demands for audiovisual speech when compared with auditory-only speech.

### Larger pupil dilation for audiovisual speech—driven by motivation or motion?

A surprising finding is that, despite agreement on the shifting of the curves, audiovisual speech leads to significantly more dilated pupils compared with auditory-only speech. The same effect is not found in frontal midline theta, despite both measures being measures of cognitive effort. There are possible explanations for why larger pupil dilation is observed in audiovisual speech—motivation is a component that drives cognitive effort, where a motivated individual could exert more effort for a task that is perceived to be more rewarding. More specifically, a more motivating task would raise the ceiling of maximal effort being applied to a task (Richter et al., 2016). Such an increase in motivation could be explained by social motivation theory (Chevallier et al., 2012), which states that social interaction holds intrinsic value such that individuals are ingrained with social motivation to orient toward biosocial signals. Biosocial signals such as face and biological motion can drive attention, leading to physiological arousal (Joshi and Gold, 2020). Indeed, larger pupil dilation has been observed for socially relevant cues where the talker would face the listener compared with facing away (Cheng et al., 2021) or when there was facial motion compared with static (Aguillon-Hernandez et al., 2020). However, social motivation cannot be the sole explanation for the larger pupil dilation observed in our results for audiovisual speech. Ricou et al. (2024) investigated features contributing to the link between pupil dilation and social motivation in visual stimuli. In their study, pupil dilation was measured across varying levels of social salience and dynamism, where the factors can be briefly summarized as the human resemblance of the target object and the degree of motion of the object, respectively. They found both factors to significantly influence pupil dilation. Specifically, pupil dilation was larger when the target object was a face when compared with a kite (social relevance) and when there was motion in the target object compared with static (even when target was a face). Supporting the notion where social relevance leads to larger pupil dilation independent of biological motion, Cheng et al. (2024) showed larger pupil dilation for audiovisual speech when the talker was upright versus inverted. In relation to our study findings, literature points toward a possible contribution of both effects due to the social relevance and movement of visual speech compared with a static face. However, due to our study design, it is not possible to dissociate the degree of contribution of either factor. Further studies controlling the amount of movement while varying the social relevance, such as inversion of the face, may allow dissociation of the two factors.

### Conclusion

In conclusion, the current study sheds more light on the influence of visual cues on the cognitive demands during speech perception. Firstly, based on the FUEL model, we assessed whether both AO and AV speech would demonstrate the inverted U-shape that characterizes effort and its interaction with motivation. We found that to be the case using both PPD and frontal midline theta power. Due to prediction that visual information lowers cognitive demands (Peelle and Sommers, 2015), we expected the point of inflection to occur at a lower SNR for AV speech. This was also shown to be the case for both outcome measures. To our knowledge, this is the first time an inverted U-shape and shift of the peak to be substantiated statistically while using neurophysiological measures associated with effort allocation. Overall, the study demonstrates the dynamic allocation of effort between AV and AO speech as a function of task demands.

## Synthesis

Reviewing Editor: Ifat Levy, Yale School of Medicine Decisions are customarily a result of the Reviewing Editor and the peer reviewers coming together and discussing their recommendations until a consensus is reached. When revisions are invited, a fact-based synthesis statement explaining their decision and outlining what is needed to prepare a revision will be listed below. The following reviewer(s) agreed to reveal their identity: Lucas Parra. Note: If this manuscript was transferred from JNeurosci and a decision was made to accept the manuscript without peer review, a brief statement to this effect will instead be what is listed below.

The authors did a nice job in addressing concerns of the previous review at JN.

## References

[B1] Aguillon-Hernandez N, Mofid Y, Latinus M, Roché L, Bufo MR, Lemaire M, Malvy J, Martineau J, Wardak C, Bonnet-Brilhault F (2020) The pupil: a window on social automatic processing in autism spectrum disorder children. J Child Psychol Psychiatry 61:768–778. 10.1111/jcpp.13170 31823380

[B2] Bates D, Mächler M, Bolker B, Walker S (2015) Fitting linear mixed-effects models using lme4. J Stat Softw 67:1–48. 10.18637/jss.v067.i01

[B3] Beatty J, Wagoner BL (1978) Pupillometric signs of brain activation vary with level of cognitive processing. Science 199:1216–1218. 10.1126/science.628837 628837

[B4] Brown VA, Strand JF (2019) About face: seeing the talker improves spoken word recognition but increases listening effort. J Cogn 2:1–20. 10.5334/JOC.89 31807726 PMC6873894

[B5] Cavanagh JF, Frank MJ (2014) Frontal theta as a mechanism for cognitive control. Trends Cogn Sci 18:414–421. 10.1016/J.TICS.2014.04.012 24835663 PMC4112145

[B6] Chandrasekaran C, Trubanova A, Stillittano S, Caplier A, Ghazanfar AA (2009) The natural statistics of audiovisual speech. PLoS Comput Biol 5:e1000436. 10.1371/journal.pcbi.1000436 19609344 PMC2700967

[B7] Cheng Y, Liu W, Yuan X, Jiang Y (2021) The eyes have it: perception of social interaction unfolds through pupil dilation. Neurosci Bull 37:1595–1598. 10.1007/s12264-021-00739-z 34212296 PMC8566692

[B8] Cheng Y, Yuan X, Jiang Y (2024) Eye pupil signals life motion perception. Atten Percept Psychophys 86:579–586. 10.3758/s13414-023-02729-x 37258891

[B9] Chevallier C, Kohls G, Troiani V, Brodkin ES, Schultz RT (2012) The social motivation theory of autism. Trends Cogn Sci 16:231–239. 10.1016/j.tics.2012.02.007 22425667 PMC3329932

[B10] Delorme A, Makeig S (2004) EEGLAB: an open source toolbox for analysis of single-trial EEG dynamics including independent component analysis. J Neurosci Methods 134:9–21. 10.1016/j.jneumeth.2003.10.009 15102499

[B11] Gelman A, Su YS (2024) arm: Data analysis using regression and multilevel/hierarchical models (Version 1.14-4) [R package]. https://CRAN.R-project.org/package=arm.

[B12] Gevins A (1997) High-resolution EEG mapping of cortical activation related to working memory: effects of task difficulty, type of processing, and practice. Cereb Cortex 7:374–385. 10.1093/cercor/7.4.374 9177767

[B13] Gieshoff AC (2021) Does it help to see the speaker’s lip movements? Transl Cogn Behav 4:1–25. 10.1075/tcb.00049.gie

[B14] Gilzenrat MS, Nieuwenhuis S, Jepma M, Cohen JD (2010) Pupil diameter tracks changes in control state predicted by the adaptive gain theory of locus coeruleus function. Cogn Affect Behav Neurosci 10:252–269. 10.3758/CABN.10.2.252 20498349 PMC3403821

[B15] Jacobs J, Hwang G, Curran T, Kahana MJ (2006) EEG oscillations and recognition memory: theta correlates of memory retrieval and decision making. Neuroimage 32:978–987. 10.1016/j.neuroimage.2006.02.018 16843012

[B16] Jensen O, Tesche CD (2002) Frontal theta activity in humans increases with memory load in a working memory task. Eur J Neurosci 15:1395–1399. 10.1046/j.1460-9568.2002.01975.x 11994134

[B17] Joshi S, Gold JI (2020) Pupil size as a window on neural substrates of cognition. Trends Cogn Sci 24:466–480. 10.1016/j.tics.2020.03.005 32331857 PMC7271902

[B18] Kadem M, Herrmann B, Rodd JM, Johnsrude IS (2020) Pupil dilation is sensitive to semantic ambiguity and acoustic degradation. Trends Hear 24:2331216520964068. 10.1177/2331216520964068 33124518 PMC7607724

[B19] Kahneman D, Tversky A (1973) On the psychology of prediction. Psychol Rev 80:237–251. 10.1037/h0034747

[B20] Koelewijn T, Zekveld AA, Festen JM, Kramer SE (2012) Pupil dilation uncovers extra listening effort in the presence of a single-talker masker. Ear Hear 33:291–300. 10.1097/AUD.0b013e3182310019 21921797

[B21] Koelewijn T, Shinn-Cunningham BG, Zekveld AA, Kramer SE (2014) The pupil response is sensitive to divided attention during speech processing. Hear Res 312:114–120. 10.1016/j.heares.2014.03.010 24709275 PMC4634867

[B22] Kraus F, Tune S, Obleser J, Herrmann B (2023) Neural α oscillations and pupil size differentially index cognitive demand under competing audiovisual task conditions. J Neurosci 43:4352–4364. 10.1523/JNEUROSCI.2181-22.2023 37160365 PMC10255021

[B23] Kressner AA, Jensen-Rico KM, Kizach J, Man BKL, Pedersen AK, Bramsløw L, Hansen LB, Balling LW, Kirkwood B, May T (2024) A corpus of audio-visual recordings of linguistically balanced, Danish sentences for speech-in-noise experiments. Speech Commun 165:103141. 10.1016/j.specom.2024.103141

[B24] Kuchinsky SE, Ahlstrom JB, Vaden KI, Cute SL, Humes LE, Dubno JR, Eckert MA (2013) Pupil size varies with word listening and response selection difficulty in older adults with hearing loss. Psychophysiology 50:23–34. 10.1111/j.1469-8986.2012.01477.x 23157603 PMC3527636

[B25] Kuznetsova A, Brockhoff PB, Christensen RHB (2017) Lmertest package: tests in linear mixed effects models. J Stat Softw 82:1–26. 10.18637/jss.v082.i13

[B26] Martin JT, Whittaker AH, Johnston SJ (2020) Component processes in free-viewing visual search: insights from fixation-aligned pupillary response averaging. J Vis 20:5. 10.1167/jov.20.7.5 PMC7424908 32634226

[B27] McGarrigle R, Munro KJ, Dawes P, Stewart AJ, Moore DR, Barry JG, Amitay S (2014) Listening effort and fatigue: what exactly are we measuring? A British society of audiology cognition in hearing special interest group ‘white paper’. Int J Audiol 53:433–445. 10.3109/14992027.2014.890296 24673660

[B28] Mishra S, Lunner T, Stenfelt S, Rönnberg J, Rudner M (2013) Visual information can hinder working memory processing of speech. J Speech Lang Hear Res 56:1120–1132. 10.1044/1092-4388(2012/12-0033) 23785180

[B29] Moradi S, Lidestam B, Danielsson H, Ng EHN, Rönnberg J (2017) Visual cues contribute differentially to audiovisual perception of consonants and vowels in improving recognition and reducing cognitive demands in listeners with hearing impairment using hearing aids. J Speech Lang Hear Res 60:2687–2703. 10.1044/2016_JSLHR-H-16-0160 28651255

[B30] Ohlenforst B, Zekveld AA, Lunner T, Wendt D, Naylor G, Wang Y, Versfeld NJ, Kramer SE (2017) Impact of stimulus-related factors and hearing impairment on listening effort as indicated by pupil dilation. Hear Res 351:68–79. 10.1016/j.heares.2017.05.012 28622894

[B31] Ohlenforst B, Wendt D, Kramer SE, Naylor G, Zekveld AA, Lunner T (2018) Impact of SNR, masker type and noise reduction processing on sentence recognition performance and listening effort as indicated by the pupil dilation response. Hear Res 365:90–99. 10.1016/j.heares.2018.05.003 29779607

[B32] Onton J, Delorme A, Makeig S (2005) Frontal midline EEG dynamics during working memory. Neuroimage 27:341–356. 10.1016/j.neuroimage.2005.04.014 15927487

[B33] Oostenveld R, Fries P, Maris E, Schoffelen J-M (2011) Fieldtrip: open source software for advanced analysis of MEG, EEG, and invasive electrophysiological data. Comput Intell Neurosci 2011:1–9. 10.1155/2011/156869 21253357 PMC3021840

[B34] Peelle JE (2017) Listening effort: how the cognitive consequences of acoustic challenge are reflected in brain and behavior. Ear Hear 39:204–214. 10.1097/AUD.0000000000000494 PMC5821557 28938250

[B35] Peelle JE, Sommers MS (2015) Prediction and constraint in audiovisual speech perception. Cortex 68:169–181; Masson SpA. 10.1016/j.cortex.2015.03.006 25890390 PMC4475441

[B36] Pichora-Fuller MK, et al. (2016) Hearing impairment and cognitive energy: the framework for understanding effortful listening (FUEL). Ear Hear 37:5S–27S. 10.1097/AUD.0000000000000312 27355771

[B37] Piquado T, Isaacowitz D, Wingfield A (2010) Pupillometry as a measure of cognitive effort in younger and older adults. Psychophysiology 47:560–569. 10.1111/j.1469-8986.2009.00947.x 20070575 PMC2867103

[B38] Raghavachari S, Lisman JE, Tully M, Madsen JR, Bromfield EB, Kahana MJ (2006) Theta oscillations in human cortex during a working-memory task: evidence for local generators. J Neurophysiol 95:1630–1638. 10.1152/jn.00409.2005 16207788

[B39] Richter M, Gendolla GHE, Wright RA (2016) Three decades of research on motivational intensity theory: what we have learned about effort and what we still don't know. Adv Motiv Sci 3:149–186. 10.1016/bs.adms.2016.02.001

[B40] Ricou C, Rabadan V, Mofid Y, Aguillon-Hernandez N, Wardak C (2024) Pupil dilation reflects the social and motion content of faces. Soc Cogn Affect Neurosci 19:nsae055. 10.1093/scan/nsae055 39167473 PMC11403811

[B41] Rönnberg J, et al. (2013) The ease of language understanding (ELU) model: theoretical, empirical, and clinical advances. Front Syst Neurosci 7:31. 10.3389/fnsys.2013.00031 23874273 PMC3710434

[B42] Sarampalis A, Kalluri S, Edwards B, Hafter E (2009) Objective measures of listening effort: effects of background noise and noise reduction. J Speech Lang Hear Res 52:1230–1240. 10.1044/1092-4388(2009/08-0111) 19380604

[B43] Seifi Ala T, Graversen C, Wendt D, Alickovic E, Whitmer WM, Lunner T (2020) An exploratory study of EEG alpha oscillation and pupil dilation in hearing-aid users during effortful listening to continuous speech. PLoS One 15:e0235782. 10.1371/journal.pone.0235782 32649733 PMC7351195

[B44] Sommers MS, Phelps D (2016) Listening effort in younger and older adults: a comparison of auditory-only and auditory-visual presentations. Ear Hear 37:62S–68S. 10.1097/AUD.0000000000000322 27355772 PMC4942117

[B45] Stolte M, Gollan B, Ansorge U (2020) Tracking visual search demands and memory load through pupil dilation. J Vis 20:21. 10.1167/jov.20.6.21 PMC7416901 32589197

[B46] ten Oever S, Schroeder CE, Poeppel D, van Atteveldt N, Zion-Golumbic E (2014) Rhythmicity and cross-modal temporal cues facilitate detection. Neuropsychologia 63:43–50. 10.1016/j.neuropsychologia.2014.08.008 25128589 PMC4209287

[B47] Tye-Murray N, Sommers M, Spehar B (2007) Auditory and visual lexical neighborhoods in audiovisual speech perception. Trends Amplif 11:233–241. 10.1177/1084713807307409 18003867 PMC4111531

[B48] Wendt D, Hietkamp RK, Lunner T (2017) Impact of noise and noise reduction on processing effort: a pupillometry study. Ear Hear 38:690–700. 10.1097/AUD.0000000000000454 28640038

[B49] Winn MB, Wendt D, Koelewijn T, Kuchinsky SE (2018) Best practices and advice for using pupillometry to measure listening effort: an introduction for those who want to get started. Trends Hear 22:2331216518800869. 10.1177/2331216518800869 30261825 PMC6166306

[B50] Wisniewski MG, Thompson ER, Iyer N, Estepp JR, Goder-Reiser MN, Sullivan SC (2015) Frontal midline θ power as an index of listening effort. Neuroreport 26:94–99. 10.1097/WNR.0000000000000306 25536119

[B51] Wisniewski MG, Thompson ER, Iyer N (2017) Theta- and alpha-power enhancements in the electroencephalogram as an auditory delayed match-to-sample task becomes impossibly difficult. Psychophysiology 54:1916–1928. 10.1111/psyp.12968 28792606

[B52] Wykowska A, Anderl C, Schubö A, Hommel B (2013) Motivation modulates visual attention: evidence from pupillometry. Front Psychol 4:59. 10.3389/fpsyg.2013.00059 23407868 PMC3569841

[B53] Zekveld AA, Kramer SE, Festen JM (2010) Pupil response as an indication of effortful listening: the influence of sentence intelligibility. Ear Hear 31:480–490. 10.1097/AUD.0b013e3181d4f251 20588118

[B54] Zekveld AA, Kramer SE, Festen JM (2011) Cognitive load during speech perception in noise: the influence of age, hearing loss, and cognition on the pupil response. Ear Hear 32:498–510. 10.1097/AUD.0b013e31820512bb 21233711

[B55] Zekveld AA, Kramer SE (2014) Cognitive processing load across a wide range of listening conditions: insights from pupillometry. Psychophysiology 51:277–284. 10.1111/psyp.12151 24506437

